# NLRP3 inflammasome, an immune‐inflammatory target in pathogenesis and treatment of cardiovascular diseases

**DOI:** 10.1002/ctm2.13

**Published:** 2020-04-09

**Authors:** Yucheng Wang, Xiaoxiao Liu, Hui Shi, Yong Yu, Ying Yu, Minghui Li, Ruizhen Chen

**Affiliations:** ^1^ Department of Cardiology Zhongshan Hospital Shanghai Institute of Cardiovascular Diseases Shanghai Medical College of Fudan University Shanghai China; ^2^ Department of General Practice Zhongshan Hospital Shanghai Medical College of Fudan University Shanghai China

**Keywords:** atherosclerosis, heart failure, inflammation, ischemia/reperfusion injury, NLRP3 inflammasome

## Abstract

Inflammation is an important process involved in several cardiovascular diseases (CVDs), and nod‐like receptor family pyrin domain containing 3 (NLRP3) inflammasome is a vital player in innate immunity and inflammation. In this review, we aim to provide a comprehensive summary of the current knowledge on the role and involvement of NLRP3 inflammasome in the pathogenesis and treatment of CVDs. NLRP3 inflammasome functions as a molecular platform, and triggers the activation of caspase‐1 and cleavage of pro‐IL‐1β, pro‐IL‐18, and gasdermin D (GSDMD). Cleaved NT‐GSDMD forms pores in the cell membrane and initiates pyroptosis, inducing cell death and release of many intracellular pro‐inflammatory molecules. NLRP3 inflammasome activation is triggered via inter‐related pathways downstream of K^+^ efflux, lysosomal disruption, and mitochondrial dysfunction. In addition, the Golgi apparatus and noncoding RNAs are gradually being recognized to play important roles in NLRP3 inflammasome activation. Many investigations have revealed the association between NLRP3 inflammasome and CVDs, including atherosclerosis, ischemia/reperfusion (I/R) injury and heart failure induced by pressure overload or cardiomyopathy. Some existing medications, including orthodox and natural medicines, used for CVD treatment have been newly discovered to act via NLRP3 inflammasome. In addition, NLRP3 inflammasome pathway components such as NLRP3, caspase‐1, and IL‐1β may be considered as novel therapeutic targets for CVDs. Thus, NLRP3 inflammasome is a key molecule involved in the pathogenesis of CVDs, and further research focused on development of NLRP3 inflammasome‐based targeted therapies for CVDs and the clinical evaluation of these therapies is essential.

**Abbreviations**
7‐Keto: 7‐ketocholesterol
ABCA1/G1: ATP‐binding cassette A1 and G1
aPC: activated protein C
ASC: apoptosis‐associated speck‐like protein containing a CARD
CaMKIIδ: Calcium/calmodulin‐dependent protein kinase II δ
CANTOS: Canakinumab Anti‐inflammatory Thrombosis Outcomes Study
ChC: cholesterol crystal
CHF: congestive heart failure
CMECs: cardiac microvascular endothelial cells
COLCOT: Colchicine Cardiovascular Outcomes Trial
CVDs: cardiovascular diseases
DAG: diacylglycerol
DAMPs: danger‐associated molecular patterns
dTGN: dispersed trans‐Golgi network
ESC: esterified cholesterol
FRC: free cholesterol
HF: heart failure
I/R: ischemia/reperfusion
IHD: ischemic heart disease
lncRNA: long noncoding RNA
NF‐κB: nuclear factor kappa B
NLRP3: nod‐like receptor family pyrin domain containing 3
oxLDL: oxidized low‐density lipoprotein
PAMPs: pathogen‐associated molecular patterns
PKD: protein kinase D
PRRs: pattern‐recognition receptors
ROS: reactive oxygen species
SCAP: SREBP cleavage‐activating protein
SREBP: sterol regulatory element‐binding protein
TAC: transverse aortic constriction
TET2: tet methylcytosine dioxygenase 2
TLR: Toll‐like receptor

## 1 BACKGROUND

Inflammation plays an important role in cardiovascular diseases (CVDs). Generally, pathogenic, metabolic, or ischemic etiologies induce the release of pro‐inflammatory cytokines such as IL‐1, IL‐6, IL‐18, and TNF‐α, and inflammatory cell infiltration. In addition, clinical studies such as the Canakinumab Anti‐inflammatory Thrombosis Outcomes Study (CANTOS) and Colchicine Cardiovascular Outcomes Trial proved that anti‐inflammatory treatment lowers the incidence of cardiovascular events.^1^, ^2^ Therefore, inflammation may be a common process involved in different CVDs, and investigating the potential of anti‐inflammatory treatment in CVDs may be the focus of future research.

Innate immune system, which includes innate immune cells and cytokines, mediates inflammation. Innate immune responses occur via different types of receptors termed pattern‐recognition receptors (PRRs), which are widely expressed on the cell surface or in intracellular vesicles and cytosol in monocytes, macrophages, neutrophils, mast cells, DC cells, and NK cells.^3^ Nod‐like receptor family pyrin domain‐containing 3 (NLRP3) inflammasome, the most thoroughly researched inflammasome type, has a PRR as sensor. NLRP3 inflammasome is a multiprotein complex mainly activated by stimuli from pathogen‐associated molecular patterns (PAMPs) and danger‐associated molecular patterns (DAMPs), and it further activates caspase‐1, which cleaves and maturates the pro‐inflammatory cytokines IL‐1β and IL‐18.^4^ Such NLRP3 inflammasome‐activated caspase‐1 also cleaves full‐length gasdermin D (GSDMD) into two parts: N‐terminal GSDMD (NT‐GSDMD) and C‐terminal GSDMD (CT‐GSDMD). NT‐GSDMD shows a lipid‐binding preference and it oligomerizes into membranes to form cell membrane pores and initializes pyroptosis, a type of programed cell death. Additionally, opening of NT‐GSDMD pore also promotes the release of cytokines and other inflammatory factors.^4^ Thus, NLRP3 inflammasome is a vital player in innate immunity and inflammation and has been widely investigated in different CVDs. Therefore, in this article, we review the current knowledge regarding the mechanisms and roles of NLRP3 inflammasome in various CVDs, and highlight the potential CVD‐treatment strategies involving the NLRP3 inflammasome pathway. This review may establish that NLRP3 inflammasome is a key regulator in the immune‐inflammatory pathway of CVDs and targeting NLRP3 inflammasome is an important strategy to explore anti‐inflammatory therapy of CVDs.

## 2 MECHANISM UNDERLYING NLRP3 INFLAMMASOME ACTIVATION

**FIGURE 1:**
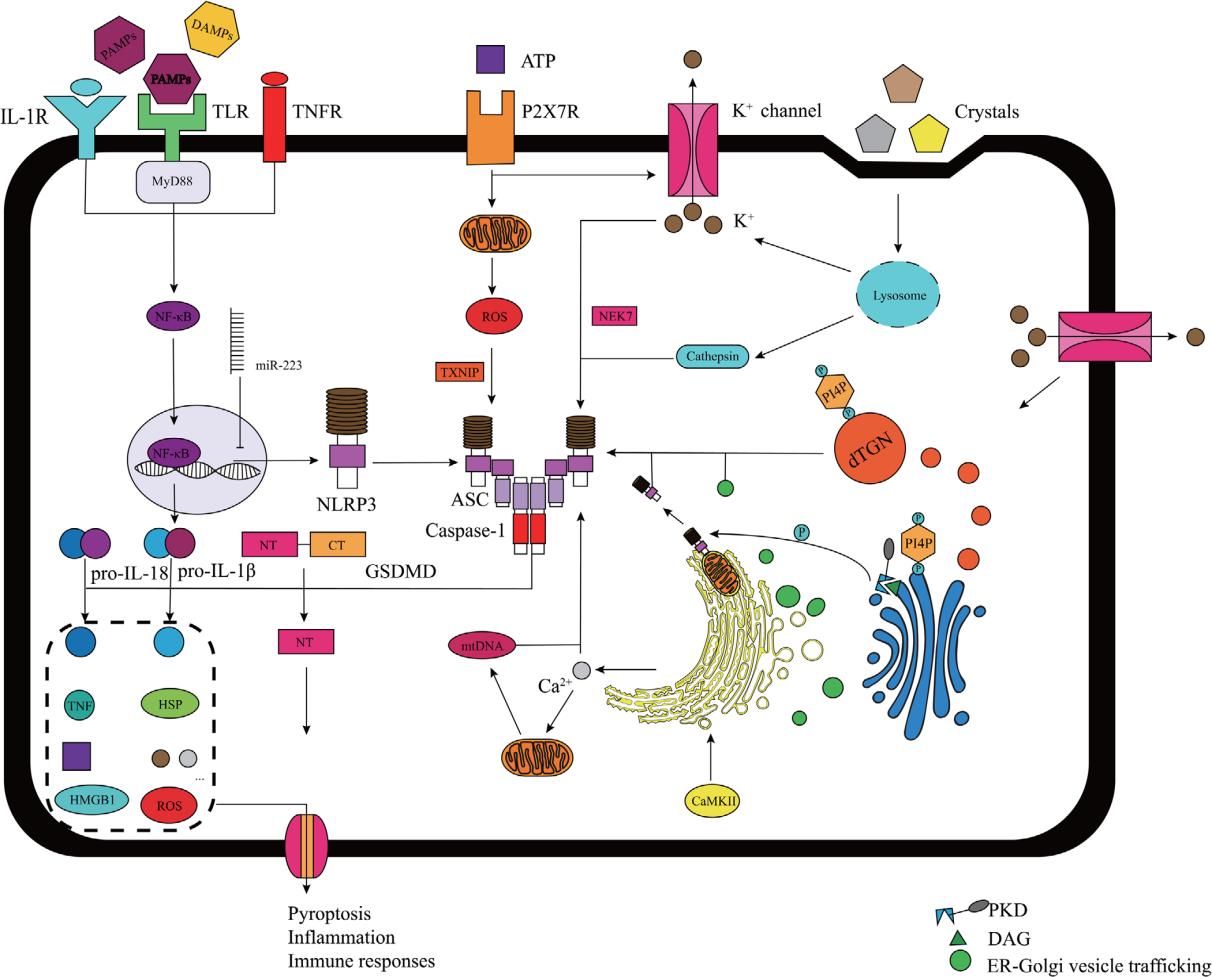
An overview of mechanisms underlying NLRP3 inflammasome activation. NLRP3 inflammasome activation occurs via two steps: priming and activating. PAMPs and DAMPs, detected by PRRs, trigger the downstream NF‐κB signaling pathway, promoting the transcription of NLRP3 and pro‐IL‐1β. However, several noncoding RNAs such as miR‐223, inhibit the transcription of NLRP3 and pro‐IL‐1β. NLRP3 inflammasome activation has lots of triggers including K^+^ efflux, Ca^2+^ mobilization, ER stress, lysosomal leakage and mitochondrial dysfunction. NLRP3 activation is also triggered via several mechanisms involving the Golgi apparatus and ER‐Golgi vesicle trafficking. Dispersed TGN provides a scaffold for NLRP3 puncta formation, promoting the assembly and activation of NLRP3 inflammasome; PtdIns4P serves as the binding site. NLRP3 inflammasome activation induces the cleavage of GSDMD, pro‐IL‐1β, and pro‐IL‐18. NT‐GSDMD forms a pore in the cell membrane, thereby inducing pyroptosis and release of intracellular factors. (DAG, diacylglycerol; DAMP, danger‐associated molecular pattern; ER, endoplasmic reticulum; GSDMD, gasdermin D; NF‐κB, nuclear factor kappa B; NT‐GSDMD, N terminal‐GSDMD; PAMP, pathogen‐associated molecular pattern; PKD, protein kinase D; PRRs, pattern‐recognition receptors; PtdIns4P, phosphatidylinositol‐4‐phosphate; TGN, trans‐Golgi network)

NLRP3 inflammasome consists of the sensor NLRP3, the adaptor apoptosis‐associated speck‐like protein containing a CARD (ASC), and the effector caspase‐1. The complete NLRP3 activation process occurs via two steps: priming and activation (Figure 1). The priming stage involves the transcriptional regulation of NLRP3 and precursor of IL‐1β. Recognition of various PAMPs and DAMPs by PRRs and stimulation of cytokines such as TNF‐α induce the nuclear translocation of nuclear factor kappa B (NF‐κB) and promote pro‐IL‐1β and NLRP3 transcription.^5^, ^6^ However, pro‐IL‐18 is constitutively expressed and IL‐18 transcription does not correlate with NF‐κB pathway activation.^4^

After the priming stage, the NLRP3 inflammasome assembly is essential for signal transduction from upstream molecules to the downstream effector molecules. Several molecular and cellular activities trigger NLRP3 inflammasome activation. These activities are categorized according to their upstream signals, and the main activities include K^+^ efflux, lysosomal disruption, and mitochondrial dysfunction. K^+^ efflux is the most noted event in NLRP3 inflammasome activation. The mechanism underlying K^+^ efflux‐mediated NLRP3 activation involves the binding of never‐in‐mitosis A‐related kinase 7 (NEK7) to NLRP3 resulting in a molecular complex formation, which is an essential intermediate step in the process of NLRP3 inflammasome activation downstream of K^+^ efflux.^7^ Lysosomal disruption is induced by both foreign and self‐derived particles such as silica, cholesterol, and uric acid crystals.^8^ Such lysosomal rupture releases cathepsins, which mediate NLRP3 inflammasome activation, and broad‐spectrum cathepsin inhibitors alleviate this action.^9^ Mitochondrial dysfunction and reactive oxygen species (ROS) release are important upstream events of NLRP3 inflammasome activation. As ROS concentration alters, thioredoxin interacting protein dissociates from thioredoxin and binds to NLRP3, inducing NLRP3 inflammasome activation.^10^ In addition, endoplasmic reticulum stress, Ca^2+^ mobilization, and some metabolic changes also serve as upstream signals for NLRP3 activation, and these pathways are interrelated.

The significant role of Golgi apparatus in NLRP3 inflammasome activation has gradually been recognized. Disruption of ER‐Golgi trafficking inhibited NLRP3 inflammasome assembly and caspase‐1 activation, indicating the involvement of ER‐Golgi vesicle trafficking in NLRP3 activation.^11^ Furthermore, NLRP3 inflammasome activation was reported to be accompanied by mitochondrial clustering around the Golgi. In nigericin‐stimulated bone marrow‐derived macrophages, diacylglycerol (DAG) enrichment in the Golgi promoted the recruitment of protein kinase D (PKD), a key downstream effector of DAG. The PKD recruited at the Golgi, phosphorylated NLRP3 leading to the release of NLRP3 from mitochondria‐associated endoplasmic reticulum membranes, and triggered the assembly of the NLRP3 inflammasome.^12^ Moreover, dispersed trans‐Golgi network (dTGN) also provides a scaffold for NLRP3 inflammasome aggregation and activation via phosphatidylinositol‐4‐phosphate, a negatively charged factor on dTGN, which functions as a binding site for NLRP3.^13^ NLRP3 inflammasome activation requires the binding of sterol regulatory element‐binding protein 2 (SREBP2) and SREBP cleavage‐activating protein (SCAP) to form the SREBP2‐SCAB complex, which is involved in cholesterol biosynthesis. NLRP3 translocates to the Golgi apparatus and forms a ternary complex with SCAP and SREBP2, thereby activating NLRP3 inflammasome.^14^ However, this research area is in the preliminary stage, and further investigation is warranted to elucidate the role of Golgi apparatus and its involvement in the NLRP3 inflammasome pathway in CVDs.

Intriguingly, noncoding RNAs, mainly microRNAs, also influence NLRP3 inflammasome activation (Table 1). Several miRNAs such as miR‐223^15^ and miR‐495‐3p^16^ affect NLRP3 transcription directly by binding to the 3′UT region of NLRP3. In addition, miRNAs such as miR‐145a‐5p,^17^ miR‐30c‐5p,^18^ miR‐383‐3p,^19^ and miR‐9‐5p^20^ impair NLRP3 inflammasome activation in CVDs by targeting CD137, FOXO3, IL‐1R2, and JAK1, respectively, all of which are upstream regulators of NLRP3 inflammasome activation. Moreover, long noncoding RNAs (lncRNAs), such as lncRNA MEG3, improve NLRP3 expression by sponging miR‐223.^21^ In conclusion, the mechanism underlying NLRP3 inflammation activation is extremely intricate and a consensus model has not yet been generated.

**TABLE 1:** Selected noncoding RNAs involving NLRP3 inflammasome and pyroptosis in several cardiovascular diseases (CVDs)

NcRNA	Target	Testify	Expression	Species	Disease	Reference
miR‐22‐3p	NLRP3	Luci	Down	Rat	Coronary heart disease	94
miR‐495‐3p	NLRP3	Luci	Down	Mouse	I/R injury	16
miR‐145a‐5p	CD137 & NFATC1	Luci	Down	Mouse	Atherosclerosis	17
miR‐30c‐5p	FOXO3	Luci	Down	Human	Atherosclerosis	18
miR‐383‐3p	IL1R2	Luci	Down	Rat	Atherosclerosis	19
miR‐9‐5p	ORP9	Luci	Down	Human	Atherosclerosis	20
miR‐125a‐5p	TET2	Luci	Up	Human	Atherosclerosis	29
miR‐21	Unknown	Caspase‐1 activity	Down	Mouse	Myocardial infarction	95
miRNA‐30d	FOXO3a	Luci	Up	Rat	Diabetic cardiomyopathy	59
lncRNA MEG3	miR‐223	Luci	Up	Human	Atherosclerosis	21
lncRNA MALAT1	miR‐23c	PCR and WB	Up	Rat	Diabetic atherosclerosis	78
lncRNA Kcnq1ot1	miR‐214‐3p/caspase‐1	Luci	Up	Mouse	Diabetic cardiomyopathy	58

Abbreviation: Luci, luciferase reporter assay.

## 3 NLRP3 INFLAMMASOME IN THE PATHOGENESIS OF CVDS

**FIGURE 2:**
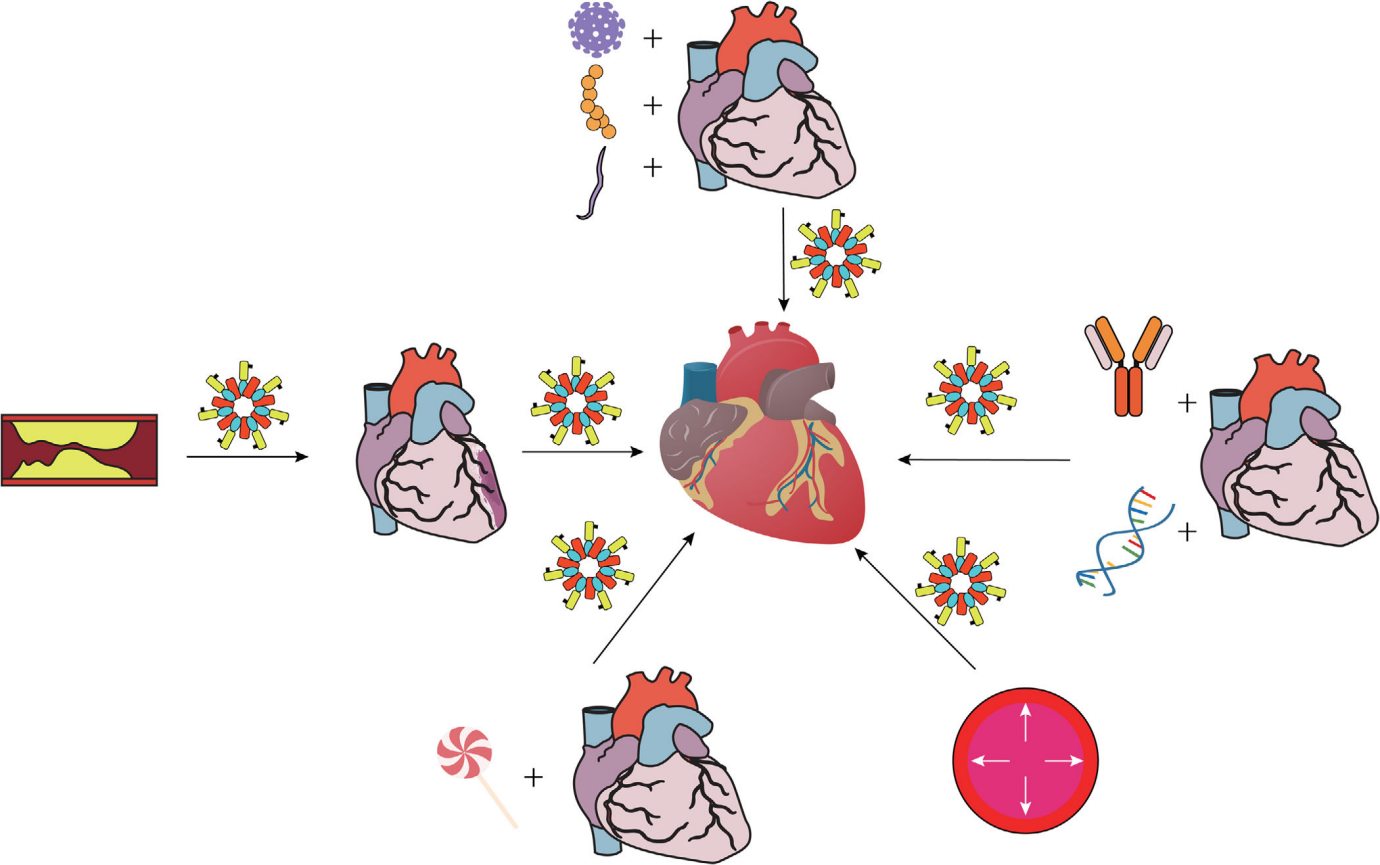
Associations between cardiovascular diseases (CVDs) involving NLRP3 inflammasome. The continuous progression of atherosclerosis significantly induces the occurrence of ischemia/reperfusion injury, whereas heart failure is the chronic turnover of ischemic heart diseases (IHDs). In addition, heart failure is a common endpoint of many other CVDs, including pressure‐induced myocardial morbidity, diabetic cardiomyopathy, infectious cardiomyopathies (induced by viruses, bacteria, and parasites), and cardiomyopathies of other etiologies (such as genetic and autoimmune). NLRP3 inflammasome plays important role in the development of these diseases

A broad‐spectrum expression of NLRP3 is observed in myeloid cells such as DCs, monocytes/macrocytes, and neutrophils, which are all constituents of the innate immunity.^22^ In addition, macrophages, DCs, neutrophils, and some other immune cells reside in the heart, whereas macrophages account for more than 70% of the resident immune cells.^23^ Other non‐immune cells such as endothelial cells (ECs) and cardiac fibroblasts are also emerging as significant participants in cardiac innate immunity. NLRP3 inflammasome activation in these cells and cardiomyocytes initiates immune inflammatory reactions and plays fundamental roles in atherosclerosis, ischemia/reperfusion (I/R) injury, and heart failure (HF) with different etiologies. We next provide a detailed account of the role and involvement of NLRP3 inflammasome in the pathogenesis of these CVDs (Figure 2).

### 3.1 NLRP3 inflammasome in atherosclerosis

Atherosclerosis, the main cause of ischemic heart disease (IHD), is a vascular pathological process characterized by the formation of atheroma, an intimal lesion that impinges and narrows the vascular lumen, causing decreased blood supply.^24^ Atherosclerosis consists of four main pathogenic events: (a) endothelial injury; (b) lipoprotein (mainly oxidized LDL and cholesterol crystal) accumulation in the vessel wall; (c) monocyte adhesion, migration, and differentiation into macrophages and foam cell formation; and (d) smooth muscle cell recruitment and proliferation. NLRP3 inflammasome is involved in some of these events during atherosclerosis pathogenesis.

#### 3.1.1 Endothelial injury

Endothelial injury or intima integrity loss is the initial step of atherosclerosis. Different types of injuries, including mechanical, immune‐mediated, and chemical‐induced injuries can lead to endothelial injury. NLRP3 inflammasome activation in ECs is a critical event in several endothelial injuries. Cigarette smoking is one of the major risk factors of atherosclerosis. Nicotine increases the expression of both NLRP3 and ASC, and triggers NLRP3 inflammasome activation and pyroptosis in human aortic endothelial cells.^25^ Pollution is another important reason for NLRP3 inflammasome activation‐mediated endothelial injury. Compared to mice exposed to filtered air, mice exposed to PM2.5 showed exacerbated atherosclerotic plaque formation and enhanced NLRP3 inflammasome activation in the aorta, as indicated by increased serum levels of IL‐1β and IL‐18.^26^ Acrolein and cadmium, two common environmental pollutants, also cause NLRP3 inflammasome‐mediated EC injury.^27^, ^28^ In addition, NLRP3 inflammasome has been reported to be involved in endothelial injury and EC loss due to metabolic etiologies, including abnormal uptake of lipids such as oxidized low‐density lipoprotein (oxLDL) and cholesterol. In fact, oxLDL treatment upregulated the protein levels of NLRP3, caspase‐1, and IL‐1β in vascular ECs in a dose‐dependent manner and increased the number of PI‐positive cells, suggestive of increased pyroptosis. Further investigation revealed that oxLDL inhibited tet methylcytosine dioxygenase 2 (TET2), which significantly downregulates the expression of caspase‐1, NLRP3, and IL‐1β by improving mitochondrial functions and blocking the NF‐κB pathway.^29^ Colocalization assays revealed that cholesterol crystal (ChC) or 7‐ketocholesterol (7‐Keto) considerably increased NLRP3 inflammasome generation and activation in mouse carotid arterial ECs.^30^ In addition, ChCs induced severe endothelial dysfunction in coronary arteries of *Nlrp3^+/+^* mice, compared with the coronary arteries of transgenic *Nlrp3^−/−^* mice.^31^ Interestingly, short‐chain fatty acids, the metabolites from gut microbes, also exert anti‐atherosclerotic effects by preserving endothelial function. A study involving partial‐ligated carotid artery mouse model showed that butyrate significantly decreased cholesterol‐induced NLRP3 inflammasome activation in the arterial walls.^32^ The protective role of butyrate involves blockage of the lipid raft redox signaling pathway and decrease in ChC‐ and 7‐Keto‐mediated production of free radicals.

#### 3.1.2 Monocyte differentiation into macrophages and engulfment of lipoproteins

After endothelial dysfunction, monocytes adhere to the lesion sites, differentiate into macrophages, and engulf lipoproteins, including oxLDL and ChCs. Such macrophages with engulfed lipids transform into foam cells. NLRP3 inflammasome activation is involved in monocyte adhesion and foam cell formation. oxLDL was reported to upregulate SREBP‐1, thereby promoting oxLDL‐induced lipid accumulation and foam cell formation. Moreover, IL‐1β, the product of NLRP3 inflammasome activation, increased the RNA and protein expression of SREBP‐1 in monocytes and monocyte‐derived macrophages.^33^ Another study evaluated foam cell formation by assessing intracellular lipid droplets, cholesterol content, and acyl‐coenzyme mRNA levels and investigated monocyte adhesion by determining the number of monocytes adhering to vascular smooth muscle cells. This study showed that NF‐κB inhibition using BMS‐345541 or NLRP3 inflammasome inhibition using MCC950 decreased oxLDL‐induced foam cell formation and monocyte adhesion.^34^ ChCs also trigger arterial inflammation and destabilization of atherosclerotic plaques. An equilibrium is maintained between esterified cholesterol (ESC) available in the sub‐intima and the free cholesterol (FRC) generated by macrophages via the action of cholesteryl ester hydrolases on ESC. However, disequilibrium between ESC and FRC affects foam cell and ChC formation. Growth of ChCs activates NLRP3 inflammasome and induces inflammation and plaque destabilization.^35^ Cholesterol efflux is mediated by the cholesterol transporters ATP‐binding cassette A1 and G1 (ABCA1/G1), which transform cholesterol into high‐density lipoprotein. Cholesterol efflux mediated by inflammasome also plays fundamental roles in atherogenesis. Bone marrow transplantation from mice with myeloid *Abca1/g1* deficiency along with deficiency of the inflammasome components *Nlrp3* or *Caspase‐1/11* into *LdIr^−/−^* mice fed with a western‐type diet showed that *Nlrp3* and *Caspase‐1/11* deficiency alleviated atherosclerotic lesions in myeloid *Abca1/g1*‐deficient *Ldlr^−/−^* mice. The study also indicated that inflammasome activation promoted neutrophil recruitment and neutrophil extracellular trap formation in plaques.^36^ Collectively, these reports indicate that accumulation of lipids such as oxLDL and ChCs induces NLRP3 inflammasome activation, which promotes monocyte adhesion, macrophage transformation into foam cells, and the subsequent development of atherosclerotic lesions. Intriguingly, microbial pathogens augment atherosclerosis via NLRP3 inflammasome. *Chlamydia pneumoniae* induced extracellular IL‐1β release, which decreased GPR109a and ABCA1 expression and cholesterol efflux and induced cholesterol accumulation and foam cell formation in *Ldlr^−/−^* mice. Conversely, *Nlrp3* knockout alleviated the *C. pneumoniae*‐accelerated atherosclerosis.^37^ Similarly, *Porphyromonas gingivalis*, a periodontal disease pathogen, interacts with PRRs, activates NLRP3 inflammasome, and promotes atherogenesis.^38^

### 3.2 NLRP3 inflammasome in cardiac I/R injury

IHD is the leading cause of mortality worldwide, and most IHD cases involve decreased blood supply secondary to obstructive atherosclerotic vascular disease.^39^ Although early reperfusion is a cure, late reperfusion is a curse for the ischemic cardiac tissues. NLRP3 inflammasome activation aggravates I/R injury, whereas inhibition of NLRP3 inflammasome significantly mitigates I/R injury, manifested by infarct size reduction. For example, caspase‐1 inhibitor, VX‐765, exerts a potent protective effect on acute myocardial infarction^40^. VX‐765 administration decreased the infarct size to 29.2 ± 4.9%, compared with 60.3 ± 3.8% in vehicles. Moreover, VX‐765 decreased blood IL‐1 levels, preserved mitochondrial complex I activity, and inhibited lactate dehydrogenase release from the heart.^40^ In Sections 3.2.1 and 3.2.1, we discuss the predominant cells and molecules involved in NLRP3 inflammasome activation during myocardial I/R injury.

#### 3.2.1 Fibroblasts: Initial activation sites for NLRP3 inflammasome in the heart

Fibroblasts are generally considered vital in the healing process after I/R injury owing to their changing phenotypes and secretion of cytokines and growth factors.^41^ However, fibroblasts also show earlier and more dramatic activities during NLRP3 inflammasome activation in response to cardiac I/R circumstances. In fact, inflammasome activation in cardiac fibroblasts was reported to be essential for myocardial I/R injury. The initial site of inflammasome activation in myocardial I/R is in cardiac fibroblasts and not cardiomyocytes. Hypoxia/reoxygenation clearly induces a considerable increase in IL‐1β production in cardiac fibroblasts but not in cardiomyocytes. Signals for inflammasome activation were also explored. ROS production and K^+^ efflux were found to mediate NLRP3 inflammasome activation in cardiac fibroblasts during I/R injury.^42^ The expression of NLRP3, IL‐1β, and IL‐18 mRNA was found to be increased in cardiac tissue post myocardial infarction, and expression in non‐cardiomyocytes altered more dramatically in an NF‐κB–dependent manner. Further investigation showed that ATP and K^+^ efflux promoted NLRP3 inflammasome assembly and IL‐1β secretion in fibroblasts, whereas NLRP3‐knockout mice showed better cardiac function and reduced infarct size.^43^ Nevertheless, more studies are required to demonstrate the role of NLRP3 activation in fibroblasts during myocardial I/R injury.

#### 3.2.2 Crucial molecules involved in NLRP3 inflammasome‐mediated cardiac I/R injury

TXNIP is a well‐known contributor of I/R injury, and increased TXNIP levels have been reported in the myocardium during I/R.^44^ Furthermore, immunoprecipitation assays showed a direct interaction between TXNIP and NLRP3, and *Txnip* knockdown decreased NLRP3 activation and infarct size in mouse myocardial tissues. Further exploration showed that I/R stimulated NLRP3 inflammasome activation in cardiac microvascular endothelial cells (CMECs) but not cardiomyocytes, and *Txnip* knockdown inhibited NLRP3 inflammasome activation in CMECs. The TXNIP‐mediated NLRP3 inflammasome activation occurred via ROS stimulation and was inhibited by the ROS scavenger EUK134.^45^ Activated protein C (aPC) exerts a cardioprotective effect on myocardial I/R injury not only via apoptosis^46^ but also via NLRP3 inflammasome.^47^ aPC treatment before or after myocardial I/R injury significantly reduced infarct size and reduced NLRP3 inflammasome activation. Kinetic in vivo analyses indicated that NLRP3 inflammasome activation preceded apoptosis in a mouse I/R model. In vitro experiments involving macrophages, cardiomyocytes, and cardiac fibroblasts showed that aPC comprehensively inhibited inflammasome activation via the proteinase‐activated receptor 1 and mammalian target of rapamycin complex 1 (mTORC1) signaling pathways.^47^ This study suggested that aPC exerts a fundamental and comprehensive effect on different kinds of cardiac resident cells in the context of NLRP3 inflammasome activation.

### 3.3 NLRP3 inflammasome in HF

HF usually refers to congestive heart failure (CHF) and is the final stage of many cardiac disorders. Cardiomyopathy and myocarditis induced by several factors are specific origins of CHF. NLRP3 inflammasome plays an important role in HF development and prognosis. A clinical trial involving 54 patients with HF reported increased ASC methylation and decreased plasma IL‐1β and ASC mRNA levels in the exercise group compared with the controls, and suggested that exercise promotes a better outcome for HF via epigenetic regulation of ASC.^48^ Furthermore, cardiac remodeling and fibrosis are the dominant mechanisms in HF development, and the involvement of NLRP3 inflammasome in these pathological processes is critical.

#### 3.3.1 HF induced by pressure overload

Calcium/calmodulin‐dependent protein kinase II δ (CaMKIIδ) plays an important role in HF pathogenesis. CaMKIIδ expression and activation were enhanced in rabbit and human HF,^49^ whereas treatment with CaMKII inhibitors (KN‐93 and AIP) improved contractility and preserved cardiac functions in the human failing myocardium.^50^ CaMKIIδ‐dependent activation of NLRP3 inflammasome mediated fibrosis during the failing heart conditions. Such CaMKIIδ‐dependent inflammasome activation occurs at an early point during angiotensin Ⅱ (AngⅡ) infusion, before the evident recruitment of macrophages. In addition, NLRP3 knockout or MCC‐mediated inhibition of NLRP3 inflammasome alleviated inflammation, indicated by decreased macrophage accumulation and fibrosis during AngⅡ infusion, which resembles the effect of CaMKIIδ deletion.^51^ Another study also revealed that CaMKIIδ deletion in cardiomyocytes attenuated pressure overload‐induced accumulation of CD68^+^ macrophages and cytokines such as IL‐1β and IL‐18. The cardiomyocytes isolated from mice subjected to cardiomyocyte‐specific CaMKIIδ deletion and transverse aortic constriction (TAC) showed decreased NLRP3 levels, caspase‐1 activity, and IL‐18 activation, indicating that CaMKIIδ activation in response to pressure overload triggers NLRP3 inflammasome activation.^52^ Somatic mutations in hematopoietic stem cells also influence HF incidence and severity via NLRP3 inflammasome. Mutation in TET2, an epigenetic regulator, was reported to promote HF, and transplantation of bone marrow with TET2‐deficient cells or conditional myeloid‐restrictive TET2 inactivation significantly augmented cardiac remodeling in mouse models of TAC‐ and chronic ischemia‐induced HF. In addition, TET2 deficiency enhanced IL‐1β expression, whereas MCC950 treatment significantly decreased HF development.^53^ Recently, increased S‐nitrosylation of muscle LIM protein (MLP) in pressure‐overloaded HF was reported to induce the formation of a complex between Toll‐like receptor 3 (TLR3) and receptor‐interacting protein kinase 3 (RIP3), thereby activating NLRP3 inflammasome and promoting the progression of myocardial hypertrophy.^54^ However, some studies have reported contradictory findings. One study showed that NLRP3 knockout accelerated the decrease of cardiac function, manifested by enhanced level of inflammation, fibrosis, and hypertrophy in animal models with pressure overload interruption, indicating that NLRP3 negatively regulated cardiac remodeling.^55^ Therefore, further investigation is warranted to explain the discordance between these studies.

#### 3.3.2 Specific HF due to cardiomyopathy and myocarditis

Cardiomyopathy literally means disease of the heart muscle. Although the etiologies of cardiomyopathy are mainly idiopathic, they may be associated with infectious, metabolic, genetic, and immune factors. Cardiomyopathy usually shows clinical symptoms similar to those of HF, which is characterized by reduced cardiac outflow, and is one of the prognostic outcomes of myocardial infarction. Cardiomyopathy and myocarditis, along with myocardial infarction, induce HF via myocardium morbidity, and NLRP3 inflammasome contributes to such myocardium morbidity‐induced HF.

Diabetic cardiomyopathy prevalence is increasing rapidly and is associated with the high morbidity rate of diabetes mellitus, which occurs due to metabolic disorders leading to inflammation, fibrosis, and cardiomyocyte death. NLRP3 inflammasome was confirmed to mediate fructose cytotoxicity in cardiomyocytes of fructose‐fed rats. Furthermore, the fructose‐fed rats and fructose‐treated H9c2 cells showed increased CD36, TLR4, TLR6, NLRP3, IL‐1β, Smad2/3 phosphorylation, and Smad4, whereas NLRP3 knockdown significantly alleviated fructose‐induced cardiac inflammation and fibrosis.^56^ In addition, H3 relaxin, an active peptide with protective roles in several CVDs, significantly inhibited NLRP3 inflammasome activation and collagen synthesis in cardiac fibroblasts under high glucose conditions. Moreover, ROS overproduction and ligand‐gated ion channel 7 (P2X7) were crucial in the NLRP3‐mediated promotion of high glucose‐induced fibrosis.^57^ Knockdown experiments using animal models of diabetic cardiomyopathy showed that noncoding RNAs, such as miRNA30d and lncRNA Kcnq1ot1, are important in mediating NLRP3 activation and pyroptosis, respectively.^58^, ^59^

Myocardial infarction, which is extremely fatal in the acute phase, can also gradually progress to chronic HF. Therefore, treatment after myocardial infarction is critical to delay HF onset. Colchicine significantly decelerated HF development after myocardial infarction, as evaluated by heart weight/tibial length, lung wet weight/dry weight ratio, and BNP expression. In particular, colchicine alleviated left ventricular remodeling and preserved contractile function. Furthermore, colchicine decreased NLRP3, ASC, and caspase‐1 levels in the MI area, thus attenuating NLRP3 inflammasome expression and activation.^60^ Intriguingly, diabetes was demonstrated to further deteriorate cardiac functions and augment HF after myocardial infarction by enhancing the activation of AIM2 and NLRC4 inflammasome instead of NLRP3 inflammasome.^61^

Several pathogens, including bacteria, viruses, and parasites cause heart diseases, and coxsackievirus B3‐induced myocarditis is the most common and important infectious heart disease. NLRP3 inflammasome is strongly associated with CVB3‐induced myocarditis. A continuous increase in the production of IL‐1β, cleaved caspase‐1, and ASC was reported in the first 7 days after CVB3 infection. Conversely, inhibiting caspase‐1 using specific inhibitor or blocking the IL‐1β pathway using a neutralizing antibody decreased the activities of myocardial enzymes, improved cardiac functions, and resulted in higher survival rate in animal models, indicating amelioration of the symptoms of CVB3‐induced myocarditis.^62^ Cathepsin B, an important regulator in the lysosome‐associated pathway of NLRP3 inflammasome activation, promotes CVB3‐induced myocarditis via activation of NLRP3 inflammasome and pyroptosis.^63^ Furthermore, in addition to cardiomyocytes, macrophages also show NLRP3 inflammasome activation in CVB3‐induced myocarditis, and instead of viral RNAs, two CVB3 capsid proteins, VP1 and VP2, trigger NLRP3 inflammasome activation in macrophages.^64^ In contrast, another study reported severe lesions, worse cardiac functions, and higher cardiac virus titers in CVB3‐infected NLRP3 knockout mice, compared with the controls.^65^

Septic cardiomyopathy is defined as acute cardiac dysfunction that occurs in patients with sepsis, and is caused by etiologies other than the generally observed cardiac etiologies. NLRP3 inflammasome activation in cardiac fibroblasts also significantly contributes to the development of septic cardiomyopathy. For example, lipopolysaccharide (LPS) treatment was reported to increase NLRP3 and IL‐1β activation in cardiac fibroblasts, and this activation was inhibited by both NLRP3 knockdown and treatment with a pharmacological inhibitor (glyburide). Interestingly, LPS‐induced activation of NLRP3 inflammasome in cardiac fibroblasts downregulated intracellular cAMP response to dobutamine in cardiomyocytes, highlighting the potential of NLRP3 inflammasome as a therapeutic target.^66^ Chagas cardiomyopathy, a parasite‐induced cardiomyopathy, is also associated with NLRP3 activation. IL‐1β and NLRP3 upregulation has been reported in patients’ indeterminate clinical form of Chagas disease.^67^ Moreover, single nucleotide polymorphism association analysis revealed that Chagas disease presenting Chagas cardiomyopathy is associated with inflammasome genes, including NLRP1 and CARD; however, NLRP3 was not investigated in this study.^68^

**FIGURE 3:**
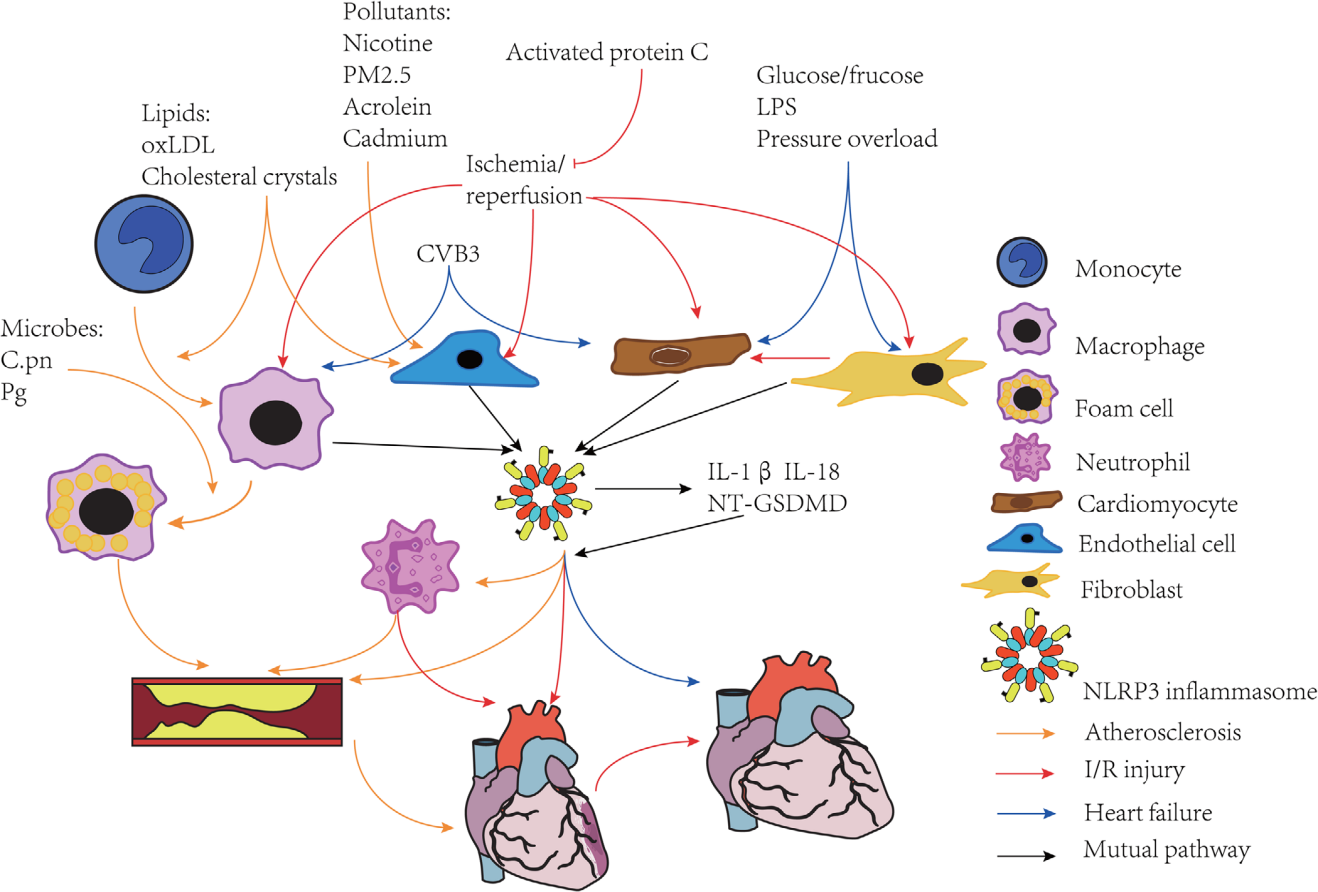
Activation of NLRP3 inflammasome and pyroptosis in multiple cells in the pathogenesis of CVDs. A, Atherosclerosis: In addition to lipids, pollutants such as nicotine, PM2.5, acrolein, and cadmium, induce endothelial injury via NLRP3 activation. Lipids also promote monocyte differentiation into macrophages, which engulf lipids and are transformed into foam cells via the NLRP3 pathway. Microbes such as *Chlamydia pneumoniae* and *Porphyromonas gingivalis* regulate this process. Neutrophils are also involved in atherosclerosis development. B, I/R injury mainly originates from coronary atherosclerosis and involves NLRP3 inflammasome activation in many cell types, including fibroblasts, cardiomyocytes, endothelial cells, and macrophages; fibroblasts are more sensitive than cardiomyocytes. NLRP3 activation also promotes the recruitment of neutrophils and further aggravates I/R injury. C, Heart failure, a common endpoint of CVDs, differs with respect to the cells wherein NLRP3 activation occurs. Glucose/fructose, LPS, and pressure overload mainly activate NLRP3 in fibroblasts and cardiomyocytes, whereas CVB3, which induces myocarditis, primarily activates NLRP3 inflammasome in macrophages and cardiomyocytes. CVB3, coxsackievirus B3; I/R, ischemia/reperfusion; LPS, lipopolysaccharide

Several other cardiomyopathies such as idiopathic dilated cardiomyopathy,^69^ genetic structural cardiomyopathy,^70^ and autoimmune myocarditis^71^ are associated with NLRP3 inflammasome activation as well. The mechanisms of all previously mentioned CVDs involving NLRP3 inflammasome and pyroptosis are summarized in Figure 3.

## 4 TARGETING NLRP3 INFLAMMASOME FOR CVD TREATMENT

Despite the availability of various treatment options, CVDs continue to pose a serious health concern. However, NLRP3 inflammasome research has suggested novel strategies for CVD treatment. The CVD treatments involving NLRP3 inflammasome can be categorized into three subsets: orthodox medication, natural medication, and novel medication (Table 2).

**TABLE 2:** Cardiovascular disease therapies targeting the NLRP3 inflammasome pathway

Drugs inhibiting the NLRP3 pathway	Category and major function	Mechanisms of action	Reference
Allopurinol	Gout drug	Suppresses NLRP3 inflammasome and alleviates fructose‐induced cardiac inflammation and fibrosis	56
Colchicine	Gout drug	Alleviates cardiac remodeling during progression of myocardial infarction to chronic heart failure via NLRP3 inflammasome	60
Cholecalciferol cholesterol emulsion	Medication to treat vitamin D deficiency in children	Inhibits pyroptosis to alleviate autoimmune myocarditis	71
Metformin	Antidiabetic drug	Alleviates fibrosis and preserves cardiac function in diabetic cardiomyopathy by inhibiting NLRP3 inflammasome and enhancing autophagy	72
Empagliflozin	Antidiabetic drug	Ameliorates cardiac hypertrophy and fibrosis in diabetic cardiomyopathy by inhibiting NLRP3 inflammasome, pyroptosis, and apoptosis	73
Pirfenidone	Medication to treat idiopathic pulmonary fibrosis	Attenuates fibrosis and ventricular remodeling in pressure‐induced heart failure by inhibiting NLRP3 inflammasome assembly and downregulates TGF‐β1	74
Statins	Hypolipidemic drugs	Alleviate oxLDL‐induced endothelial injury and diabetic cardiomyopathy by inhibiting NLRP3 activation	75, 76
Cardiac glycosides	Cardiotonic drug	Activate NLRP3 inflammasome and cause the adverse effect of promoting lactate dehydrogenase release and IL‐1β expression	77
Sinapic acid	Herbal compound	Inhibits NLRP3 inflammasome and pyroptosis via miRNA‐23 sponging by lncRNA‐MALAT1	78
Guanxinning	Traditional Chinese drug	Inhibits myocardial neutrophil infiltration, improves cardiac functions, and downregulates cleaved caspase‐1 activity and IL‐1β maturation	79
Naoxintong	Chinese Materia Medica standardized product	Decreases infiltration of pro‐inflammatory macrophages (M1 macrophages) and neutrophils in ischemia/reperfusion injury, and downregulates serum IL‐1β	80
Emodin	Laxative in traditional Chinese medicine	Inhibits cardiomyocyte pyroptosis to exert anti‐inflammatory effects in ischemia/reperfusion injury	81
Cinnamaldehyde	Herbal compound	Suppresses NLRP3 inflammasome and alleviates fructose‐induced cardiac inflammation and fibrosis	56
*Coriolus versicolor*	Mushroom compound	Ameliorates inflammation and fibrosis by inhibiting the NLRP3 inflammasome and TGF‐β1/Smad signaling pathways in diabetic heart	82
Gypenosides	Traditional Chinese drug	Decreases pyroptosis, production of IL‐1β, and cleaved caspase‐1 in diabetic cardiomyopathy	83
Total flavonoids (TA)	Herbal compound	Decreases production of IL‐1β, TNF‐α, IL‐6, and SOD, alleviates ischemia/reperfusion injury by inhibiting NLRP3 inflammasome	96
Triptolide (TP)	Herbal compound	Inhibits NLRP3 and ASC expression, inhibits NLRP3 inflammasome assembly, attenuates fibrosis via TGF‐β/Smad signaling	97
Luteolin	Herbal compound	Inhibits NLRP3 inflammasome via the TLR4/NF‐κB pathway in ischemia/reperfusion injury	98
MCC950	Novel agent	Specifically inhibits NLRP3 inflammasome and induces conformational change from active to inactive state	84, 85
CY‐09	Novel agent	Blocks NLRP3 inflammasome by directly binding to NACHT domain to inhibit NLRP3 ATPase activity	86
14,2‐(2‐Chlorobenzyl)‐N‐(4‐sulfamoylphenethyl) acrylamide	Synthetic inhibitor	Inhibits NLRP3 ATPase in a concentration‐dependent manner	87
OLT1177 (dapansutrile)	Novel agent	Small‐molecule inhibitor of NLRP3 inflammasome; significantly reduces myocardial infarction size	99
INF4E	Novel agent	A synthetic inhibitor of NLRP3 inflammasome; alleviates ischemia/reperfusion injury	100
Withaferin A	Novel agent	Blocks NF‐κB pathway and ER stress	88
Kaempferol	Novel agent	Indirectly inhibits NLRP3 inflammasome by regulating autophagy	89
Canakinumab	Antibody	Neutralizing IL‐1β antibody, significantly decreases high‐sensitive C‐reactive protein and lipid levels and attenuates inflammation	1, 90
VX‐765	Novel agent	Caspase‐1 inhibitor	40

### 4.1 Orthodox medication

Orthodox medication includes drugs that are widely used in current clinical practices to treat CVDs or other diseases. Many studies revealed that some well‐known drugs function by mediating NLRP3 activation and pyroptosis. Allopurinol, a xanthine oxidase inhibitor mainly used for gout treatment, reduced cardiac inflammation, and fibrosis, thereby ameliorating heart injury in fructose‐induced metabolic syndrome by significantly suppressing NLRP3 inflammasome activation.^56^ Colchicine, another gout medicine, significantly alleviated cardiac remodeling, improved survival, and prevented the progression of myocardial infarction to chronic HF via NLRP3 inflammasome regulation.^60^ Furthermore, a randomized controlled trial in patients with recent myocardial infarction revealed significantly lower risk of ischemic cardiovascular events in the patients treated with 0.5 mg colchicine daily, compared to that in the placebo‐treated patients.^2^ Cholecalciferol cholesterol emulsion, a commercialized drug used to treat vitamin D deficiency in children, alleviated autoimmune myocarditis via pyroptosis inhibition in mice.^71^ Metformin and empagliflozin, two well‐known antidiabetic drugs, inhibited NLRP3 inflammasome and decelerated the development of diabetic cardiomyopathy but via different mechanisms. Metformin promotes the AMPK phosphorylation,^72^ whereas empagliflozin suppresses the sGC‐cGMP‐PKG pathway.^73^ Pirfenidone, the first approved small‐molecule drug for the treatment of idiopathic pulmonary fibrosis, decreased myocardial fibrosis and ventricular remodeling by inhibiting NLRP3 inflammasome formation.^74^ Statins, the metabolism regulators, target oxLDL‐induced NLRP3 inflammasome activation in ECs and have been suggested to play a protective role against I/R injury.^75^ In addition, rosuvastatin showed a protective effect against diabetic cardiomyopathy via NLRP3 inflammasome inhibition.^76^ In contrast, cardiac glycosides, a group of broadly used cardiotonic drugs, show an adverse effect involving NLRP3 inflammasome. Interestingly, digoxin showed a specific cytotoxic effect on iPS cardiomyocytes but not on immune cells.^77^ Thus, these drugs may improve metabolism and alleviate inflammation by targeting NLRP3 inflammasome to protect the cardiovascular system.

### 4.2 Natural medication

Natural medicines such as herbal drugs and compounds isolated from natural materials, is another large category of medicines used for CVD treatment involving NLRP3 inflammasome. Several herbal drugs used for the treatment of acute ischemic CVDs are known to exert a protective effect via NLRP3 inflammasome. Sinapic acid, a compound isolated from a traditional herb, alleviated NLRP3 inflammasome activation and pyroptosis in both in vitro and in vivo models of atherosclerosis. In addition, the inhibitory role of sinapic acid in suppression of NLRP3 inflammasome occurs via miRNA23c sponging by lncRNA‐MALAT1, as confirmed using knockdown experiments involving pcDNA‐MALAT1 and MALAT1 siRNA.^78^ Guanxinning, a class B protective traditional Chinese drug widely used in various CVDs, inhibited myocardial neutrophil infiltration, improved cardiac functions, and downregulated cleaved caspase‐1 activity and IL‐1β maturation.^79^ Naoxintong, a Chinese Materia Medica standardized product, showed a similar effect on I/R injury.^80^ Emodin, a laxative used in traditional Chinese medicine, showed anti‐inflammatory effects via cardiomyocyte pyroptosis inhibition and attenuated myocardial I/R injury via the TLR4/MyD88/NF‐κB/NLRP3 inflammasome pathway.^81^ Cinnamaldehyde, a compound commonly found in specific plant species, reduced fructose‐induced cytotoxic effect in the myocardium by suppressing NLRP3 inflammasome activation via the CD36‐mediated TLR4/6‐IRAK4/1 signaling pathway.^56^ Additionally, *Coriolus versicolor*, an edible mushroom with medicinal value, significantly ameliorated inflammation and fibrosis by inhibiting the NLRP3 inflammasome and TGF‐β1/Smad signaling pathways in a rat model of diabetes mellitus.^82^ Gypenosides, used in traditional Chinese medicine, alleviated the development of diabetic cardiomyopathy by inhibiting ROS‐induced NLRP3 activation.^83^ Thus, it is essential to identify more such existing medicines, including natural drugs, that act via NLRP3 inflammasome and to investigate their therapeutic potential against different types of CVDs.

### 4.3 Novel medication

The search for novel treatment approaches is a constant pursuit in medical research and practice. Several new targets and medicines for CVD treatment are being identified owing to the intensive investigation on the NLRP3 inflammasome pathway. The targets of NLRP3 inflammasome‐mediated treatment approaches may include inhibition of NLRP3 inflammasome, IL‐1β, and caspase‐1; however, detailed investigation is essential to confirm the therapeutic effects in a clinical setting. MCC950, a well‐known NLRP3 inflammasome inhibitor, is not only widely used in research but also has potential clinical utility. MCC950 specifically blocks NLRP3 inflammasome activation without affecting the NLRP1, NLRC4, and AIM2 inflammasomes.^84^ The inhibitory effect of MCC950 on NLRP3 highlights its anti‐inflammatory potential that may be useful in many diseases. Furthermore, MCC950 induces a conformational switch in NLRP3 from the active state to an inactive state.^85^ Another small‐molecule inhibitor, CY‐09, specifically blocks NLRP3 inflammasome by directly binding to the NACHT domain to inhibit NLRP3 ATPase activity.^86^ A synthetic compound, 14,2‐(2‐chlorobenzyl)‐N‐(4‐sulfamoylphenethyl) acrylamide, may also inhibit NLRP3 ATPase in a concentration‐dependent way^87^. In addition, withaferin A and kaempferol inhibit NLRP3 via indirect mechanisms. Withaferin A is an NF‐κB inhibitor and it also blocks ER stress, affecting both NLRP3 priming and activation.^88^ In contrast, kaempferol decreases NLRP3 expression by regulating the autophagy pathway.^89^ These NLRP3 inflammasome‐targeting molecules have potentially significant clinical value but need to be investigated in further detail to ensure their applicability in the clinical setting.

Inhibition of cytokine IL‐1β was another strategy used to combat CVDs and was explored in CANTOS, which investigated the efficacy of canakinumab, a neutralizing IL‐1β antibody, in preventing the incidence of CVDs. This study revealed that canakinumab potentially reduced the recurrence of cardiovascular events in stable patients with CVD.^90^ Subsequently, a larger clinical trial conducted to investigate the effect of canakinumab in anti‐inflammatory treatment reported that canakinumab significantly decreased high‐sensitive C‐reactive protein and lipid levels and ameliorated the clinical symptoms; however, IL‐1β inhibition also increased the incidence of fatal infection. Therefore, the recommended dose of canakinumab is 150 mg every 3 months.^1^ Conversely, the IL‐1 receptor antagonist anakinra showed a long‐term effect in patients with ST‐elevated myocardial infarction but reduced inflammatory markers only within 14 days in patients with non‐ST elevated myocardial infarction.^91^, ^92^ Furthermore, significant residual inflammatory effect of IL‐18 and IL‐6 was reported after canakinumab administration for anti‐atherothrombosis treatment, indicating the requirement for developing or identifying novel inhibitors of other pro‐inflammatory cytokines such as IL‐18 and IL‐6.^93^ Therefore, novel inhibitors targeting other molecules are continuously being explored; for example, the caspase‐1 inhibitor, VX‐765, was reported to provide long‐term effects in alleviating I/R injury and preserving cardiac functions.^40^

Detailed investigation of NLRP3 inflammasome may enable the development of new strategies for the CVD treatment or enable the discovery of new clinical applications of the existing therapies. However, in addition to the continuous effort for identification of new compounds, studies focused on evaluating the efficacy of the identified compounds in the clinical setting are essential.

## 5 CONCLUSION

Activation of NLRP3 inflammasome is critical for the development of many cardiovascular disorders. The NLRP3 inflammasome plays distinct roles in different CVDs; however, because the etiology, pathological characteristics, onset and offset of these diseases are different, the inhibition of NLRP3 inflammasome and related pathways could be a convergent strategy for the treatments of many CVDs. Such novel therapies involving NLRP3 inflammasome have high potential for applicability in the clinical setting. In addition, inhibition of the NLRP3 inflammasome pathway for CVD treatment could be a new mechanism of many existing medicines. Although detailed research is essential to completely understand the role of NLRP3 inflammasome in the pathogenesis and identify novel CVD therapies, NLRP3 inflammasome may be considered as a crucial target of the immune‐inflammatory pathway and may broaden therapeutic field in CVDs.

## AUTHOR CONTRIBUTIONS

Yucheng Wang wrote the manuscript. Hui Shi and Xiaoxiao Liu provided the conception and designing. Yong Yu, Ying Yu, and Minghui Li contributed to literature search and designing. Ruizhen Chen supervised and revised the manuscript. All authors read and approved the final manuscript.

## FUNDING INFORMATION

National Natural Science Foundation of China (No. 81772109, 81521001, 81671937)

## CONFLICT OF INTEREST

The authors declare that they have no conflict of interest.
